# All-Oral Low-Dose Chemotherapy TEPIP is Effective and Well-Tolerated in Relapsed/Refractory Patients With Aggressive B-Cell Lymphoma

**DOI:** 10.3389/fonc.2022.852987

**Published:** 2022-05-10

**Authors:** Matthias A. Fante, Mona Felsenstein, Stephanie Mayer, Michael Gerken, Monika Klinkhammer-Schalke, Wolfgang Herr, Martin Vogelhuber, Albrecht Reichle, Daniel Heudobler

**Affiliations:** ^1^ Department of Internal Medicine III, Hematology and Internal Oncology, University Hospital Regensburg, Regensburg, Germany; ^2^ Bavarian Cancer Registry, Regional Centre Regensburg, Bavarian Health and Food Safety Authority, Regensburg, Germany; ^3^ Tumor Center - Institute for Quality Management and Health Services Research, University of Regensburg, Regensburg, Germany; ^4^ Bavarian Cancer Research Center (BZKF), University Hospital Regensburg, Regensburg, Germany

**Keywords:** relapsed/refractory DLBCL, metronomic chemotherapy, all-oral treatment, low-dose chemotherapy, aggressive B-cell lymphoma

## Abstract

**Purpose:**

Treatment options in patients (pts.) with advanced relapsed and refractory aggressive B-cell lymphoma are limited. Palliative all-oral chemotherapy regimens reduce in-patient visits and contribute to quality of life. The all-oral low-dose chemotherapy regimen TEPIP comprises the conventional chemotherapy agents trofosfamide, etoposide, procarbazine, idarubicin and prednisolone.

**Methods:**

Safety and efficacy of TEPIP was evaluated in an observational retrospective, single-center study at the University Medical Center Regensburg between 2010 and 2020. Treatment with TEPIP was applied for 7 or 10 days during a 28-days period. In a subgroup of fit and therapy-motivated pts. rituximab was added. End points were overall survival (OS) and progression free survival (PFS). Adverse events ≥ CTCAE grade III were reported.

**Results:**

35 highly pre-treated pts. with aggressive B-cell lymphoma were enrolled. Median age at TEPIP start was 67 years and 85% of pts. received TEPIP as ≥ third treatment line. Overall response rate (ORR) was 23% (CR 17%). Pts. benefited from additional rituximab administration (ORR 67%) and a lower number of pre-treatments (ORR 41%). The OS was 3.3 months (m) with a 1y-OS of 25.7% and the PFS amounted to 1.3 m with a 1y-PFS of 8.8%. OS and PFS were significantly prolonged in pts. that responded to treatment or additionally received rituximab. Adverse events were mainly hematological and occurred in 49% of pts.

**Conclusion:**

TEPIP was well-tolerated and induced respectable response in a difficult-to-treat patient cohort. In particular, the all-oral administration enables out-patient use with palliative intent.

## Introduction

The aggressive diffuse large B cell lymphoma (DLBCL) is the most common subtype of non-Hodgkin lymphoma (NHL) in the Western hemisphere with an incidence of 7 cases per 100,000 people (1–3) accounting for 2.8% of all cancer diagnoses worldwide (4).

While the majority of patients achieves durable and complete remission with first-line treatment (5–7), 40% do not respond or experience relapse (R/R DLBCL), most frequently within the first 2 years (8) but also later (9). Eligibility for second-line options strongly depends on the patient’s performance status and age as potentially curative standard second-line regimens comprise salvage chemotherapy followed by consolidation with high dose chemotherapy (HDT) and autologous stem cell transplantation (ASCT) (10). To undergo ASCT, a complete (CR) or at least partial remission (PR) from a previous salvage regimen is favorable, but may be only achieved in up to 60% of patients resulting in a transplantation rate of 50% (11–13). Patients failing to respond to first salvage therapy or relapsing after ASCT have a poor prognosis. However, long-term survival may be achieved by switching salvage therapy to enable subsequent ASCT (14) or by proceeding to a reduced intensity conditioning (RIC) and allogeneic transplantation (alloTx) (15).

Due to insufficient response to chemotherapy, comorbidities, and age, a substantial proportion of patients is not eligible for HDT and transplantation. During the last years, substantial progress has been made in the development of novel approaches for relapsed/refractory disease including CD19 CAR T-cells. CAR T-cell therapies achieve impressive objective response rates (ORR) of up to 74% in heavily pre-treated patients of all ages but are associated with high costs and potentially lethal adverse events such as neurotoxicity, cytokine release and protracted immunodeficiency (16–19). Contraindications to and relapse after CAR T-cell therapy or alloTx strongly limit the probability of a sustained response (20). In this context, about 50% of patients develop a relapse of DLBCL after CAR T-cell therapy representing a big therapeutic challenge (21, 22). In these palliative situations, therapies must focus on symptom control as well as prolongation and quality of life.

Remaining treatment options comprise conventional regimens with acceptable toxicity such as R-GemOx (23, 24), R-DHAOx (25), as well as R-Bendamustin (26) improving OS especially in combination with the anti-CD79b drug-conjugate polatuzumab-vedotin (27). Further, monotherapies or combinations of novel targeted agents like bi-specific antibodies (28), ibrutinib (29) and lenalidomide in combination with rituximab (30) or the anti-CD19 antibody tafasitamab (31) have been approved recently or are undergoing evaluation.

In contrast to conventional chemotherapies aiming for a maximal tolerated dose (MTD), metronomic regimens combine regularly administered, low-dose agents to reduce toxicity and overcome acquired resistance while equally targeting tumor cells and the tumor-promoting microenvironment (32–36). Until today, only few groups have focused on metronomic regimens in relapsed and refractory lymphoma, however with partially impressive results (37–39). In sum, these insights underline the urgent need for further development of well-tolerated out-patient treatment options for CAR therapy and transplantation ineligible or relapsed patients with R/R DLBCL to improve palliation.

In this retrospective, single-center study we demonstrate efficacy and safety data of an all-oral, prolonged low-dose chemotherapy regimen TEPIP (trofosfamide, etoposide, procarbazine, idarubicin, prednisolone), which was developed and administered at the University Hospital Regensburg. TEPIP is also given at doses below MTD with the goal to exert alternative antitumor effects while minimizing side effects. However, due to the rather prolonged interval breaks, this regimen (given 7 or 10 days of a 28-day cycle) does not fulfill the strict definition of a metronomic schedule.

## Methods

In this single-center study we retrospectively analyzed safety and efficacy of an all-oral chemotherapy, TEPIP, administered in 35 patients at the University Medical Center Regensburg (UKR) between 2010/01/01 and 2020/12/31. The cohort was identified by an in-clinic, medical file database query using the terms “TEPIP” and “R-TEPIP” and only aggressive B cell lymphomas were considered. All data (histologic diagnoses, clinical parameters, outcome measures) were obtained from medical reports. Due to the retrospective nature of this study and the out-patient drug administration, the data set is partially limited. The analysis was approved by the local Ethics Committee (reference number: 20-1901-104) and performed in compliance with the current Helsinki Declaration. All patients alive gave written informed consent for publication.

### Disease Classification

The underlying disease was defined as *primary* in patients with only aggressive B-cell lymphoma (DLBCL, EBV+, prolymphocytic/lymphoblastic). In contrast, the disease was termed *non-primary*, if transformed from a previous indolent lymphoma or concurrently showing aggressive and indolent histology. Lymphoma disease was staged before TEPIP start according to the Ann Arbor criteria and prognosis was assessed at first diagnosis by use of the international prognostic index (IPI) score. In cases without a reported IPI score, appropriate parameters (LDH, age, performance status, extranodal manifestations, stage) were collected to raise a definitive or in cases of incomplete data sets an “at least” IPI score in every patient. Only extranodal manifestations of the aggressive, but not of possibly concurrent indolent lymphoma were considered in the calculation of the IPI score.

### Chemotherapy Regimen

**Figure 1:**
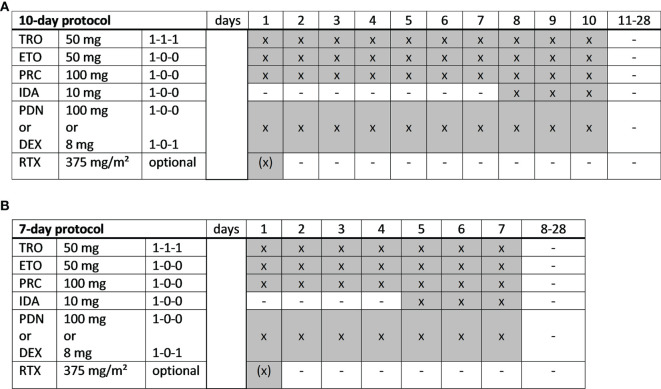
TEPIP schedules. **(A)** A full-dose, 10-day or **(B)** a dose-reduced, 7-day protocol was applied depending on the patient´s performance status and expected toxicity tolerance. TRO, trofosfamide; ETO, etoposide; PRC, procarbazine; IDA, idarubicin; PDN, prednisolone; DEX, dexamethasone; RTX, rituximab. x = administration; hyphen = no administration.

TEPIP was administered as an all-oral low-dose chemotherapy regimen allowing a full out-patient treatment. TEPIP comprises trofosfamide 150 mg (3 single doses of 50 mg), etoposide 50 mg (one single dose), procarbazine 100 mg (one single dose) and prednisolone 100 mg (one single dose) or dexamethasone 16 mg (2 single doses of 8 mg) at days 1 to 10 of 28, which was shortened to 7 days in case of numerous pre-treatments as necessary in most of the patients. On days 8 to 10 (10-day course) and 5 to 7 (7-day course), respectively, a daily single dose of idarubicin 10 mg was added (**Figure 1**). Dose reductions due to adverse events were performed according to physician´s choice. Recommendations for dose reductions included limiting trofosfamide to two or one dose of 50 mg per day, procarbazine to 50 mg per day or etoposide to 50 mg every other day. 9 patients additionally received the monoclonal anti-CD20 antibody rituximab (375 mg/m² intravenously, iv) on day 1 of each course (**Figure 1**). The course was repeated each 28 days provided that the leukocyte count exceeded 3000 per microliter and continued until disease progression or intolerability due to toxicity. Apart from an appropriate antiemesis (e.g., metoclopramide) no specific supportive therapy (e.g., granulocyte-colony-stimulating factor) was administered.

### Response Assessment

Response was reported as the best response documented by medical reports. In patients providing available CT or PET-CT imaging, response was assessed according to the 2014 Lugano criteria (40)and the recommended staging schedule was eight weeks. The objective response rate (ORR) was defined as the sum of patients acquiring complete or partial remission. Survival was analyzed as overall survival (OS) covering the period between onset of TEPIP treatment and the patient´s death or end of the observation period (2020/12/31), respectively, and progression free survival (PFS) representing the period between treatment start and diagnosis of disease progression or death due to any reason.

### Adverse Event Assessment

Toxicities are listed as reported by out-patient reports and graded according to the Common Toxicity Criteria for Adverse Events (CTCAE) version 5.0, with only adverse events of CTCAE ≥ grade III being reported in this analysis.

### Statistics

Analyses are performed by PRISM 9 (GraphPad, San Diego, CA, USA) and SPSS 28 (IBM, Armonk, NY, USA). The endpoints of the study are OS and PFS which are depicted as Kaplan-Meier curves. Differences between subgroups are determined by the Fisher´s exact test (ORR) and Mantel-Cox log-rank test (OS and PFS). Additionally, the log-rank Hazard Ratio (HR) is reported. Data are judged to be statistically significant when p < 0.05.

## Results

### Patient Characteristics

The cohort comprised 24 male (68%) and 11 female (32%) patients with a median age at first diagnosis of 63 years (range: 40 – 78 years). 23 patients (66%) suffered from primary aggressive lymphoma, including two double-hit lymphomas (both with c-myc + BCL-2 translocations), one EBV positive plasmoblastic lymphoma, one primary mediastinal large B-cell lymphoma and one prolymphocytic/lymphoblastic B-CLL (**Table 1**). 12 cases (34%) were defined as non-primary, as they either transformed from indolent lymphomas (follicular 4/12, CLL 3/12, undetermined 1/12), or showed histological characteristics of aggressive and indolent lymphomas (4/12). A subtype and cell-of-origin (COO) analysis was not available in a representative number of enrolled patients. At first diagnosis, disease was advanced (stage III-IV) in 25 patients (71%, no report: 1 pt.) with 19 patients (54%, no reports: 2 pts.) having extranodal manifestations of aggressive lymphoma. The IPI score at first diagnosis was assessable in 20 patients (57%) with 12/20 being high, 2/20 high-intermediate (HI), 2/20 low-intermediate (LI) and 4/20 low. Extensive medical report recherche allowed a labeling of remaining patients according to the IPI score with “at least HI risk” in 3 patients, resulting in a high-risk group of 17 patients (49%) with an ensured high and high-intermediate IPI score. Median time between initial diagnosis and start of TEPIP therapy was 12 months (range 0 – 163). Prior to TEPIP, median number of pre-treatments was 3 (range 0 – 6) with 1 patient (3%) receiving in 1^st^ line, 4 patients (11%) in 2^nd^ line, 12 patients (34%) in 3^rd^ line and 18 patients (51%) in 4^th^ or subsequent line. TEPIP was administered in 5 patients (14%) after high-dose chemotherapy with ASCT and 3 (9%) rituximab-naive patients (2/3 wereCD20-negative, 1/3 received ofatumumab instead of rituximab). Best response to any prior treatment lines was a complete or partial remission in 27 patients (79%), whereas 7 patients (21%) only reached a stable disease or were progressive on all prior chemotherapies. Only 3 patients (9%) responded to the previous therapy line resulting in a median PFS (previous therapy) of 1 month (range: 0 – 38).

**Table 1:** Patient characteristics at the initiation of TEPIP treatment.

	N (%)
Total	35 (100)
Sex (male)	24 (68)
Age (years):	
median (range)	67 (41 – 82)
≤ 60 years	7 (20)
60-74 years	22 (63)
≥ 75 years	6 (17)
Performance status (ECOG):	
0-1	15 (43)
≥ 2	12 (34)
no report	8 (23)
Ann Arbor stage:	
limited (stage I-II)	8 (23)
advanced (stage III-IV)	24 (68)
no report	3 (9)
LDH levels:	
normal	6 (17)
elevated	26 (74)
no report	3 (9)
IPI score	
high + high intermediate	22 (63)
low + low intermediate	8 (23)
no report	5 (14)
Lymphoma pathology	
primary aggressive	23 (66)
non-primary aggressive	12 (34)
TEPIP treatment/prior treatment	
1^st^ line	1 (3)
2^nd^ line	4 (11)
3^rd^ line	12 (34)
4^th^ or subsequent line	18 (51)
prior ASCT	5 (14)
prior rituximab	32 (91)
Response to prior lines	
sensitive (CR and PR)	27 (77)
refractory (SD and PD)	7 (20)
no prior line	1 (3)
Response to directly preceding line	3 (9)
Prior etoposide	9 (26)

IPI, international prognostic index; ASCT, autologous stem cell transplantation; CR, complete remission; PR, partial remission.

### TEPIP Treatment

At treatment start of TEPIP, median age was 67 years (range: 41 – 82 years) with 80% of patient being > 60 years and equal parts of patients in normal (ECOG 0-1) and reduced (ECOG 2-4) performance status (no report: 8 pts.) (**Table 1**). Disease was limited (stage I-II) in 8 (23%), advanced (stage III-IV) in 24 (68%) and not reported in 3 cases. 26 patients (74%) showed extranodal lymphoma manifestations (no report: 1 pt.). A median of one TEPIP course (range: 1 – 15) was administered corresponding to a duration of treatment of 1.3 months (range: 0 – 21.9 months) and 16 patients (46%) received at least 2 courses. The overall number of administered TEPIP courses in 35 patients was 91. Only 4 patients (11%) met the requirements for the 10-day chemotherapy, while 22 patients (63%) were treated *a priori* with a shortened 7-day regimen (no report: 9 pts.). Anti-CD20 antibody rituximab was added to the therapy protocol of 9 patients (26%, no reports: 2 pts.) of whom no patient was rituximab-naïve.

### Outcome and Survival Analysis

The median follow-up was 3.3 months (range 0.9 – 80.3) and at the time of analysis 33 patients had died (94%).

The best response was complete remission in 6 (17%, 1 patient with simultaneous local irradiation), partial remission in 2 (6%), stable disease in 1 (3%) and progressive disease in 26 patients (74%) corresponding to an objective response rate (ORR) of 23%. In patients receiving at least 2 TEPIP courses every second patient achieved measurable response including 38% complete remissions. ORR was significantly higher for patients who were pre-treated with less than 3 lines (41% vs. 6%), were older than 67 years (44% vs. 0%) and additionally received rituximab (67% vs. 4%). However, neither patients with chemosensitive disease (22% vs. 29%), nor with low IPI at first diagnosis and TEPIP start (17% vs. 18% and 13% vs. 23%) or limited disease at TEPIP start (25% vs. 21%) showed a significantly higher ORR. In contrast, 2 of 7 patients refractory to prior treatment lines responded to TEPIP treatment.

Therapy was discontinued due to disease progression (26/35), reduced performance status (2/35) and infectious adverse events (2/35). Of note, 5/35 patients discontinued treatment with complete remission, of which 2/5 patients received 2 and 3 courses of TEPIP, respectively, after best response, whereas in 3/5 patients the treatment was stopped immediately at diagnosis of CR. In addition, 3/5 patients received one or two courses of rituximab for maintenance therapy after complete remission and TEPIP discontinuation. Relapse in patients who discontinued TEPIP with CR occurred after a median time of 7.2 months (range: 1.5 – 54.1 months). Besides, in one patient treatment was interrupted with complete remission, started again 3 months later when relapse occurred and finished with progressive disease after initial response within 5 further courses. Subsequent next line chemotherapy was initiated in 14/35, and radiation in 3/35 patients, whereas 15/35 patients did not receive any further treatment (no report 3 pts.).

**Figure 2:**
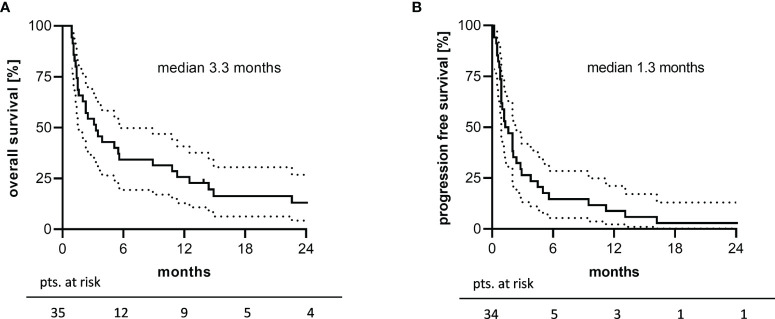
2-years survival analysis of the study cohort. Kaplan-Meier plots of **(A)** overall survival and **(B)** progression free survival are depicted. Unavailable PFS data in one patient explain the difference in patients at risk. The dotted line represents the 95% confidence interval.

Overall survival (OS) was assessable in all patients, whereas the date of progression lacked in one patient due to the out-patient setting. Median OS (mOS) was 3.3 months (**Figure 2A**) with a one- and two-year OS rate of 25.7% (95% CI: 12.8-40.8) and 13.1% (95% CI: 4.3-26.8), respectively. The median PFS (mPFS) amounted to 1.3 months (**Figure 2B**) with a one-year PFS rate of 8.8% (95% CI: 2.3-21.1). Remarkable 12.5 months mOS and 3.8 months mPFS were observed in 16 patients who received at least 2 TEPIP courses.

**Figure 3:**
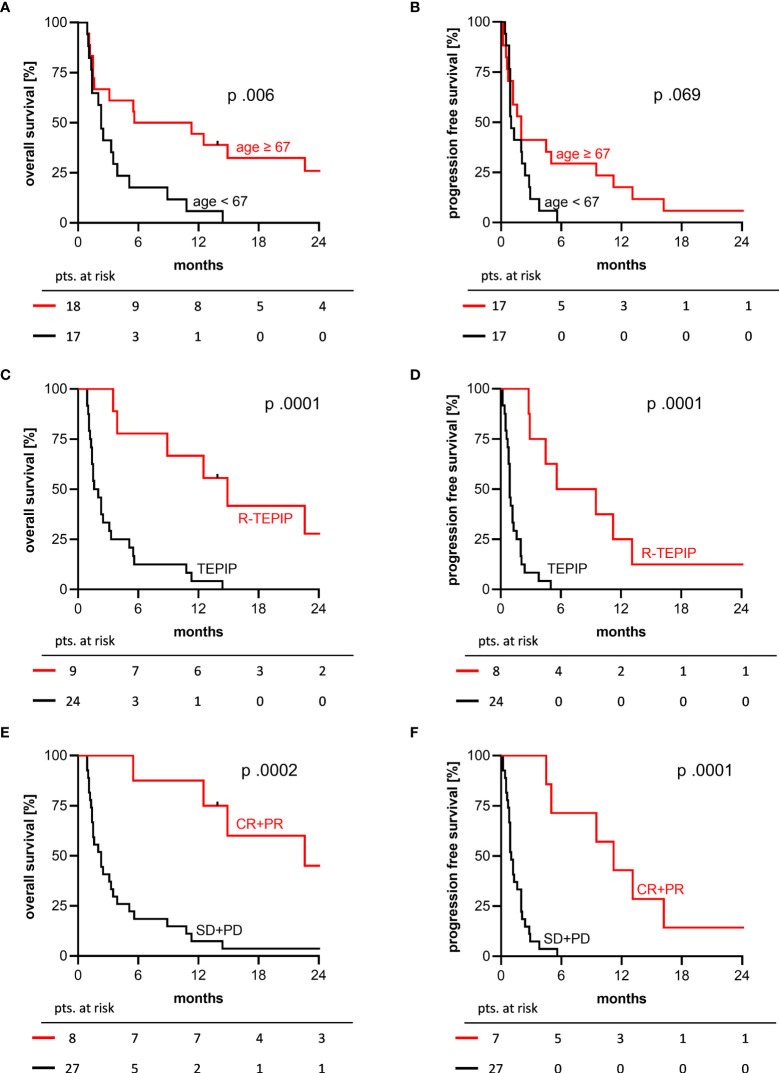
2-years OS and PFS Kaplan-Meier curves by selected patient characteristics. **(A, B)** older (red) versus younger (black) half of the study cohort, median age 67 years; **(C, D)** combination with rituximab (red) versus TEPIP alone (black); **(E, F)** responding (red) versus not responding patients (black). p values were determined by log-rank test.

Further, the older half of patients being ≥ 67 years had a significantly longer OS (median 5.6 vs. 2.3 months) but not PFS (median 2.0 vs. 1.0; *p*.069) (**Figures 3A, B**). OS and PFS were significantly longer in patients treated with a combination of rituximab + TEPIP (median 14.9 vs. 1.6 months) (**Figures 3C, D**). In responding patients (CR or PR), median duration of treatment was 4.5 months (range: 2.0 – 21.9) and only 2 patients subsequently developed disease progression under TEPIP treatment, whereas 5 patients finished treatment without evidence of residual disease (no report: 1 pt.). The median OS and PFS in this cohort were 22.6 (1y-OS 7/8 pts.; 2y-OS 3/8 pts.) and 11.2 months (1y-PFS 3/7 pts.; 2y-PFS 1/7 pts.), respectively (**Figures 3E, F**). Of note, in 6 patients reaching a CR the median OS was 37.6 months with an estimated 1- and 2-year OS of 6/6 and 3/6 pts. and 1- and 2-year PFS of 3/6 and 1/6 pts., which stands in contrast to the median OS and PFS of 2.3 and 1.0 months in patients not responding to the TEPIP treatment.

No significantly longer OS and PFS were seen in patients with a low(-intermediate) IPI score at first diagnosis and TEPIP start, an objective response to prior treatment, a limited stage at TEPIP start (Ann Arbor I/II) and a primary lymphoma ontogeny. Patients with less than 3 prior treatment lines had a numerically, but not significantly improved OS of 5.5 months (median 5.5 vs. 2.5 months, *p*.662). A detailed synopsis of the outcome and survival analysis is shown in **Table 2**.

**Table 2:** Response in patients receiving TEPIP.

	N	ORR/CR [%]	*p (ORR)*	median OS months [95%CI]	*HR [95%CI]*	*p (OS)*	median PFS months [95%CI]	*HR [95%CI]*	*p (PFS)*
**total**	**35**	**23/17**		**3.3 [1.4-5.2]**			**1.3 [0.5-2.1]**		
≥ 2 TEPIP courses	16	50/38		12.5 [0.0-28.8]			3.8 [1.7-5.9]		
IPI low/low intermed. 1^st^ dx	6	17/17	.999	2.3 [0.0-14.3]	*0.7 [0.3-1.8]*	.480	1.3 [0.4-2.2]	*0.9 [0.4-2.2]*	.795
IPI high/high intermed. 1^st^ dx	17	18/12		2.5 [1.0-4.0]			0.9 [0.7-1.1]		
IPI low/low intermed. TEPIP	8	13/13	.999	2.0 [0.0-4.8]	*1.2 [0.5-2.7]*	.704	1.3 [0.3-2.3]	*1.0 [0.5-2.5]*	.840
IPI high/high intermed. TEPIP	22	23/14		2.3 [0.1-4.5]			1.2 [0.8-1.6]		
< 3 prior treatment lines	17	41/29	**.016**	5.5 [0-15.5]	*0.9 [0.4-1.7]*	.662	2.0 [0.0-5.1]	*0.6 [0.3-1.2]*	.111
≥ 3 prior treatment lines	18	6/6		2.5 [0.8-4.2]			1.2 [0.6-1.8]		
CR/PR prior treatments	27	22/19	.999	3.5 [2.1-4.9]	*0.6 [0.2-1.7]*	.271	1.3 [0.4-2.2]	*0.9 [0.4-2.2]*	.830
SD/PD prior treatments	7	29/14		2.3 [0.2-4.4]			1.0 [0.7-1.3]		
patients < 67 years	17	0	**.003**	2.3 [1.6-3.0]	*2.3 [1.1-4.9]*	**.006**	1.0 [0.6-1.3]	*1.7 [0.9-3.5]*	.069
patients ≥ 67 years	18	44/33		5.6 [0.0-17.7]			2.0 [0.9-3.1]		
best response CR	6	–	–	37.6 [6.3-68.9]	*0.3 [0.1-0.5]*	**.0004**	11.2 [6.9-15.5]	*0.3[0.1-0.5]*	**<.0001**
best response CR+PR	8	–	–	22.6 [3.7-41.5]	*0.3 [0.1-0.5]*	**.0002**	11.2 [6.8-15.6]	*0.3 [0.1-0.5]*	**<.0001**
non-responder	27	–	–	2.3 [1.1-3.5]			1.0 [0.7-1.3]		
TEPIP with rituximab	9	67/56	**.001**	14.9 [9.0-20.8]	*0.3 [0.1-0.5]*	**.0001**	5.6 [0.0-12.5]	*0.3 [0.1-0.5]*	**<.0001**
TEPIP w/o rituximab	24	4/0		1.6 [0.9-2.3]			0.9 [0.7-1.1]		
Ann Arbor I/II at TEPIP start	8	25/25	.999	2.0 [0.0-4.5]	*0.9 [0.4-2.1]*	.858	1.3 [0.3-2.3]	*0.9 [0.4-1.8]*	.679
Ann Arbor III/IV at TEPIP start	24	21/13		2.5 [0.7-4.3]			1.2 [0.7-1.7]		
primary aggressive lymphoma	23	17/17	.402	3.3 [1.7-4.9]	*0.9 [0.4-1.8]*	.702	2.0 [0.8-3.2]	*0.9 [0.4-1.9]*	.819
non-primary aggressive lymphoma	12	33/17		2.3 [0.0-6.7]			1.2 [0.6-1.8]		

Demonstrated is the best ORR, OS and PFS with treatment. *p values* comparing different patient characteristics were determined by Fisher´s exact (ORR) or log-rank test (OS and PFS).N, number of patients; HR, hazard ratio; ORR, overall response rate; OS, overall survival; PFS, progression free survival; CI, confidence interval; IPI, international prognostic index; 1^st^ dx, first diagnosis; CR, complete remission; PR, partial remission; SD, stable disease; PD, progressive disease; w/o, without. Bold values represent significant (as opposed to nonbold, nonsignificant) test results.

### Safety Analysis

Since all patients received TEPIP on an out-patient basis, analysis of treatment-related adverse events was limited. To the best of our knowledge, 17/35 patients (49%) developed in total 30 adverse events ≥ CTCAE grade III, whereas no events were reported in 16/35 patients (46%, no report: 2 pts., **Table 3**). Grade IV toxicities only occurred in hematologic adverse events (4 pts. with grade IV neutropenia/leukopenia, 2 pts. with grade IV thrombopenia). Of note, the proportion of elderly patients (≥67 years) who noticed a side effect was equal to the subgroup of younger patients (9/18, 50%). The most frequently observed events (17/35) were hematologic findings with leukopenia/neutropenia in 8 patients, anemia in 6 patients and thrombopenia in 3 patients (**Table 3**). Unfortunately, there are no reliable data on the use of G-CSF and erythrocyte or platelet concentrates. Infectious complications occurred in 6/35 patients with two patients suffering from pneumonia, one patient from fever of unknown origin and one patient of urinary tract infection. Two patients were hospitalized with a septic event, of whom one subsequently died (**Table 3**). Therapy was discontinued due to infectious adverse events only in 2 patients. Gastrointestinal complications were reported in 5 patients with nausea and diarrhea in two and intestinal hemorrhage in two patients. Of note, one patient experienced a not lethal intestinal perforation, possibly caused be a preliminary mesenterial lymphoma manifestation. Rare adverse events observed each in one patient were pulmonary embolism and an epileptic seizure (**Table 3**). One patient died of a massive oropharyngeal tumor bleeding after the first chemo course, which was, however, not considered therapy-related.

**Table 3:** Adverse events ≥ CTCAE grade III occurring during the treatment of TEPIP.

	N (%)
**Hematologic**	**17 (49)**
Anemia	6 (17)
Leukopenia/Neutropenia	8 (23)
Thrombopenia	3 (9)
**Non-hematologic**	**13 (37)**
*Infections*	*6 (17)*
- pneumonia	2 (6)
- UTI	1 (3)
- FUO	1 (3)
- sepsis	2 (6)
*Gastrointestinal AE*	*5 (14)*
- nausea/diarrhea	2 (6)
- intestinal hemorrhage	2 (6)
- intestinal perforation	1 (3)
*Others*	*2 (6)*
- pulmonary embolism	1 (3)
- epileptic seizure	1 (3)

UTI, urinary tract infection; FUO, fever of unknown origin; AE, adverse events.

## Discussion

Patients suffering from relapsed and refractory aggressive B-cell lymphoma have poor clinical outcomes and limited response rates to next line therapy, posing a global challenge to physicians and often requiring palliative treatment (14, 20, 41). Even more, there is an urgent medical need for out-patient therapies that are well-tolerated without lacking efficacy. In this retrospective study, we present robust safety and efficacy data of the locally developed, all-oral, low-dose chemotherapy regimen TEPIP, which has been administered to 35 patients with palliative intent at the University Hospital Regensburg over the past decade. The regimen analyzed here consists of 4 orally available drugs plus steroids that have been reported to achieve objective response in lymphoma disease with concomitant tolerable safety profiles [trofosfamide (42–45), etoposide (37–39, 46), procarbazine (37, 47), idarubicin (48, 49)].

In our heavily pretreated patient population having received a median of 3 prior lines, we observed an ORR of 23% (17% CR). Median survival was 3.3 months with 26% of patients surviving the first year and 9% remaining progression free during this period. Larger prospective (randomized) trials investigating a similar patient population showed comparable response rates. Pettengell et al. reported a complete response rate of 20% for pixantrone treatment vs. 5.7% for comparator chemotherapy as salvage therapy in patients with aggressive lymphoma and a median of 3 previous therapy lines (50). By minimizing the number of patient visits all-oral therapies contribute to quality of life. In this context, several trials have investigated the use of orally administered lenalidomide (25 mg on days 1–21 of 28-day cycles) in heavily pre-treated patients suffering from DLBCL. The NHL-002 and NHL-003 trials showed ORR rates of 19 and28% with CR rates (including CR unconfirmed) of 12 and 7% (51, 52). Taking into account that in the TEPIP cohort only 3 patients (9%) (**Table 1**) had an objective response to the directly preceding therapy, the ORR of 23% (17% CR) is even more notable. Moreover, with the tight median TEPIP treatment duration of 1.3 months it must be noted, that the intention to treat was highly palliative in a large proportion of patients. Consistently, 16 patients who received at least two courses of TEPIP had significantly improved median OS and PFS of 12.5 and 3.8 months, respectively, and an ORR of 50% (CR 38%). Additional administration of rituximab to the regimen in 9 fit and treatment-motivated patients resulted in a further improved ORR of 67% with prolonged OS (14.9 months) and PFS (5.6 months), however requiring in-patient treatment. Of note, in patients responding to treatment (CR or PR) OS and PFS was significantly prolonged, which highlights the potential for a sustained treatment effect. Interestingly, despite its relevance to good prognosis (53, 54), low and low-intermediate IPI score did not result in better ORR, OS, and PFS at either initial diagnosis or TEPIP initiation.

Patient age over 60 years contributes to the IPI score and is a risk factor for poor prognosis in DLBCL (53, 54). Besides a decreased treatment tolerance due to comorbidities, also alterations in drug metabolism and impaired bone-marrow capacity may preclude the administration of intensive (salvage) chemotherapy in the elderly and frail (55–57). The TEPIP cohort had a median age of 67 years, indicating a vulnerable patient population. Nevertheless, also elderly patients (≥67 years) benefited from TEPIP treatment in terms of median OS (5.6 months) and PFS (2.0 months). However, it should be noted that in patients ≥67 years the regimen was started earlier in treatment (median 2 vs. 3 prior lines), administered in a higher number of courses (median 1.5 vs. 1), and more frequently combined with rituximab (6 vs. 3 pts.).

Treatment-emergent adverse events CTCAE grade III or higher occurred in every second patient and were predominantly hematologic (49%). This could be at least partially explained by an assumable reduction of the bone marrow capacity in our highly pre-treated cohort. However, recommendations for dose-adjustment apparently prevented the translation of cytopenia into non-hematologic events, as only 17% of patients developed infections and 6% of patients had bleeding complications ≥ CTCAE grade III. Remarkably, only in 2 patients the treatment was discontinued due to infectious complications. As dose-adjustment was performed according to the physician´s choice, reliable numeric statements are not available. Older patients did not experience a higher number of adverse events ≥ CTCAE grade III (50%). Due to retrospectivity and the out-patient setting additional unreported events cannot be completely excluded. However, compared with previous palliative regimens in R/R DLBCL patients (24, 29, 38, 50, 58) and given the seniority of the multiply pretreated cohort, the safety profile of TEPIP is tolerable and manageable. For instance, a phase 3 trial treating a similar patient population with pixantrone vs. comparator chemotherapy showed grade 3 or 4 toxicity in 76.5%vs 52.2% with neutropenia being the predominant event (50).

Several novel strategies, including CAR-T cells (16, 17), bi-specific (28) and further antibodies [tafasitamab (58), polatuzumab-vedotin (27)] have achieved impressive and sustained responses in the challenging subset of R/R DLBCL patients. Nevertheless, the use of these state-of-the-art therapies is limited due to patient comorbidities, but also in the global context due to high costs and availability. In addition, most novel drugs are administered intravenously and require close monitoring of side effects or hospitalization. However especially in advanced palliative settings, all-oral therapies are sought to prolong survival while maximizing quality of life. Besides, our oral low-dose regimen TEPIP was developed to provide a decentralized therapy concept for rural areas such as eastern Bavaria.

To date, few studies have evaluated the safety and efficacy of such-like therapies in DLBCL. Coleman et al. reported a well-tolerated, metronomically applied low-dose regimen PEP-C, that achieved remarkable overall response rates of 69%, albeit predominantly in indolent lymphoma (37). Another all-oral metronomic combination of cyclophosphamide, etoposide and prednisolone used in aggressive lymphoma yielded comparable response rates to TEPIP (CR 19.4 vs. 17%) while median OS (18.8 months) and PFS (10.5 months) distinctly exceeded the results presented here. However, in this study, the median treatment duration was 6 months (TEPIP 1.3 months), and 77% of patients received treatment as first or second line (TEPIP 14%) (39). A recently published prospective Italian trial demonstrated efficacy (ORR 71%) and safety of an all-oral regimen DEVEC resulting in 1-year OS and PFS of 48 and 39%, respectively, despite an old and multiply pretreated population with R/R DLBCL (38). In contrast to the TEPIP trial, the median number of courses administered was higher (median 6 vs. 1 course) and 49% of patients also received rituximab (TEPIP 26%), both factors which have been shown to significantly improve the effect of TEPIP treatment.

While the PEP-C as well as the DEVEC regimen comprise cyclophosphamide as an alkylating agent, the TEPIP schedule uses trofosfamide which might not be available in other countries. Trofosfamide (oral drug) was choosen because it is approved for the use in R/R NHL in Germany. In contrast, the oral form of cyclophosphamide is only approved in breast cancer and auto-immune disease. Since trofosfamide is an oxazaphosphorine that is mainly metabolized to ifosfamide and to a smaller extent to cyclophosphamide, replacing trofosfamide by cyclophosphamide might be possible. Taking the efficacy of PEP-C and DEVEC or of continuous oral cyclophosphamide into account, a replacement strategy seems to be feasible and potentially also effective. However, we do not have any data on the use of cyclophosphamide instead of trofosfamide in the TEPIP regimen.

We acknowledge the limitations of the study presented here. In addition to a partially incomplete data set due to the palliative out-patient setting and the retrospective analysis without a control group, the relatively small number of patients treated and the short median duration of TEPIP administration negatively affect the explanatory power of the results. Additionally, rituximab which has shown to synergistically enhance the effect of all-oral regimens was administered in only a small number of patients. Nevertheless, in elderly patients and patients who received at least two courses and/or a combination with rituximab ORR and survival were remarkable, which underscores the value of TEPIP therapy in the palliative out-patient care of R/R DLBCL patients. Further evaluation in larger cohorts and randomized trials is needed to gain further experience.

## Data Availability Statement

The raw data supporting the conclusions of this article will be made available by the authors, without undue reservation.

## Ethics Statement

The studies involving human participants were reviewed and approved by Ethics Committee University of Regensburg (reference number: 20-1901-104). All patients provided their written informed consent to participate in this study.

## Author Contributions

MAF, SM, WH, MV, AR and DH treated the patients. MAF, MF, MG, MK-S and DH analyzed the data. MAF, AR and DH wrote the manuscript. All authors revised the manuscript critically, approved the final manuscript, and agreed to be accountable for all aspects of the manuscript.

## Conflict of Interest

The authors declare that the research was conducted in the absence of any commercial or financial relationships that could be construed as a potential conflict of interest.

## Publisher’s Note

All claims expressed in this article are solely those of the authors and do not necessarily represent those of their affiliated organizations, or those of the publisher, the editors and the reviewers. Any product that may be evaluated in this article, or claim that may be made by its manufacturer, is not guaranteed or endorsed by the publisher.
